# NWE: Node-weighted expansion for protein complex prediction using random walk distances

**DOI:** 10.1186/1477-5956-9-S1-S14

**Published:** 2011-10-14

**Authors:** Osamu Maruyama, Ayaka Chihara

**Affiliations:** 1 Institute of Mathematics for Industry, Kyushu University, 744 Motooka Nishi-ku Fukuoka 819-0395, Japan; 2 Graduate School of Systems Life Sciences, Kyushu University, 744 Motooka Nishi-ku Fukuoka 819-0395, Japan

## Abstract

**Background:**

Protein complexes are important entities to organize various biological processes in the cell, like signal transduction, gene expression, and molecular transmission. In most cases, proteins perform their intrinsic tasks in association with their specific interacting partners, forming protein complexes. Therefore, an enriched catalog of protein complexes in a cell could accelerate further research to elucidate the mechanisms underlying many biological processes. However, known complexes are still limited. Thus, it is a challenging problem to computationally predict protein complexes from protein-protein interaction networks, and other genome-wide data sets.

**Methods:**

Macropol *et al.* proposed a protein complex prediction algorithm, called RRW, which repeatedly expands a current cluster of proteins according to the stationary vector of a random walk with restarts with the cluster whose proteins are equally weighted. In the cluster expansion, all the proteins within the cluster have equal influences on determination of newly added protein to the cluster. In this paper, we extend the RRW algorithm by introducing a random walk with restarts with a cluster of proteins, each of which is weighted by the sum of the strengths of supporting evidence for the direct physical interactions involving the protein. The resulting algorithm is called NWE (Node-Weighted Expansion of clusters of proteins). Those interaction data are obtained from the WI-PHI database.

**Results:**

We have validated the biological significance of the results using curated complexes in the CYC2008 database, and compared our method to RRW and MCL (Markov Clustering), a popular clustering-based method, and found that our algorithm outperforms the other algorithms.

**Conclusions:**

It turned out that it is an effective approach in protein complex prediction to expand a cluster of proteins, each of which is weighted by the sum of the strengths of supporting evidence for the direct physical interactions involving the protein.

## Background

Protein complexes are important entities to organize various biological processes in the cell, like signal transduction, gene expression, and molecular transmission. In most cases, proteins perform their intrinsic tasks in association with their specific interacting partners, forming protein complexes. Therefore, an enriched catalog of protein complexes in a cell could accelerate further research to elucidate the mechanisms underlying many biological processes. However, known complexes are still limited. Thus, it is a challenging problem to computationally predict protein complexes from protein-protein interaction (PPI) networks, and other genome-wide data sets.

Many high-throughput techniques (such as yeast-two-hybrid) have enabled genome-wide screening of pairwise PPIs (see [1-5] for example). Those identified PPIs are accumulated into databases like DIP [6] and BioGRID [7], which are increasing in size. Those accumulated PPI data make it more important to develop more efficient and accurate intelligent tools for the identification of protein complexes from such PPI data.

It is known that densely connected subgraphs of a PPI network are often overlapped with known protein complexes [8]. Based on this observation, a large number of global clustering algorithms are proposed for protein complex prediction, like MCL [9], SPC and MC [8], MCODE [10], RNSC [11], and PCP [12]. Supervised learning approaches are also investigated by Qi *et al.*[13] and Maruyama [Maruyam, O: Heterodimeric Protein Complex Identification, submitted for publication, 2011]. They construct Bayesian classifiers from positive and negative examples of protein complexes and applied them to protein complexes prediction. The extracted features are shown to be capable to distinguish complex versus non-complexes. Recently, a survey paper on computational approaches for protein complex prediction appeared and compared performance of many of the above mentioned algorithms [14]. This article is useful to discuss further research directions in the problem of protein complex prediction.

A *random walks with restarts* or *repeated random walk* on a graph is a kind of a random walk, in which at every tick time, a random walker has a chance to get back to one or more start nodes from any current node with a fixed, common and constant probability [15-17]. Let *C* be a set of start nodes, which can be a singleton set. The result of a random walk with restart with *C* is the stationary probabilities from *C* to all the nodes of the given graph. These probabilities can be considered to be the affinity or proximity from *C* to individual nodes. They are exploited to predict protein complexes in [15,16] with |*C*| = 1 and [17] with |*C*| *≥* 1.

**Figure 1:**
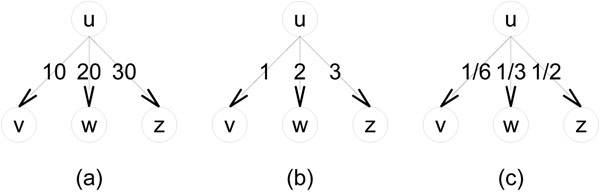
**The original weights of WI-PHI are lost** (a) shows a graph with relatively high weights, and (b) gives a graph with relatively low weights. (c) represents the same resulting transition probabilities of the graphs of (a) and (b).

It is known that, in general, the random walk technique exploits the global structure of a network by simulating the behavior of a random walker [18]. By introducing the restart mechanism to a simple random walk, a local structure centered around the start node is intensively reflected in the resulting stationary probabilities. Namely, the restart mechanism makes the local structure of a start node biased. This feature will make it more promising the approach of a random walk with restarts in protein complex prediction. Note that MCL [9] also carries out a kind of a random walk but not a random walk with restarts. What the algorithm carries out is an alternate repeat of the two processes of a simple random walk and an inflation. Macropol *et al.*[17] proposed a protein complex prediction algorithm, called RRW, which repeatedly expands a cluster of proteins according to the stationary probabilities of a random walk with restarts with the cluster, where an input PPI network is assumed to be a graph whose edges are weighted by the strength of supporting evidence for functional association. RRW is reported to outperform MCL, though MCL is known to be the most outperforming algorithm in the performance comparison papers [19,20]. Here is a critical problem on RRW. The PPI network that RRW used in the work [17] is WI-PHI, [21], a genome-wide PPI network of *S. cerevisiae*, which is obtained by integrating various heterogeneous data sources of PPIs. Thus, the weight of an interaction in WI-PHI is used as the weight of the corresponding edge of the PPI network. However, inside the algorithm of RRW, these weights of PPIs are transformed into the transition probability matrix of the given PPI network. Namely, in this transformation, original information about the given weights of PPIs are lost. An example is illustrated graphically in Figure 1. The graph in (a) has relatively high weights and the graph in (b) has relatively low weights. The corresponding transition probabilities of them are the same, which is shown in (c). Therefore, RRW can lose much information of the original PPI weights of a given PPI network.

Our motivation of this work is to devise a protein complex prediction algorithm that can exploit the original PPI weights of a given PPI network as much as possible. In this paper, we propose the method called *node-weighted expansion* of clusters of nodes to predict protein complexes, which can be attained by extending the RRW algorithm. The random walk we use here is a random walk with restarts with a cluster whose nodes are *non-uniformly weighted*. Our method repeatedly expands a cluster, *C*, of nodes to a larger one by adding the node to which the resulting random walk distance from *C* is the highest among all the nodes except ones in *C*. Our implementation of this method is called NWE (Node-Weighted Expansion for protein complex prediction).

The biological significances of the predicted clusters by NWE are validated by comparison with manually curated heteromeric protein complexes of *S. cerevisiae* in the CYC2008 database [22]. In performance comparison of NWE with RRW and MCL, NWE performs better than the others, even on noisy input networks. Thus we can conclude that the node-weighted expansion method yields improvement in protein complex prediction. We have also examined the coverage of a predicted cluster by a gene ontology (GO) term. The result shows that even a predicted cluster which does not overlap with any known complexes in CYC2008 often obtains a high coverage. Thus, some of the predicted clusters are expected to be true protein complexes.

## Methods

In this section, we describe the materials, the problem we address here, and our method for the problem.

### Materials

WI-PHI [21] is an integrated interaction network derived from various heterogeneous data sources of protein-protein interactions. The underlying input PPI network on which we simulate a random walk with restarts is derived from WI-PHI. This database is a list of protein-protein interactions with 50 000 interactions over 5 955 proteins of yeast. Each interaction has a weight, which is determined from various heterogeneous data sources, including results of tandem affinity purification coupled to MS (TAP-MS), large-scale yeast two-hybrid studies, and small-scale experiments stored in dedicated databases. CYC2008 [22] is a comprehensive catalog of 408 curated protein complexes. We use those complexes of size four or more as gold standards in the evaluation of the predicted clusters. The number of them is 149.

### Problem of predicting protein complexes

Let *G* = (*V,E*) be an undirected graph representing a protein-protein interaction network, where *V* be the set of nodes, representing proteins, and *E* is the set of weighted undirected edges, where the weight of an edge should be a positive real and is supposed to show the strength, reliability, and so on, of the corresponding PPI. In this work, this graph is derived from WI-PHI. The problem we address in this study is stated as follows: Given a protein-protein interaction network, the protein complex prediction problem is defined as the problem of finding a set of statistically significant clusters of proteins. The matching statistics of predicted clusters are calculated with the protein complexes in the CYC2008 database. A predicted cluster which does not share any common protein with any of the gold standard complexes can be good candidates for new protein complexes because CYC2008 will be not a complete list of yeast protein complexes.

### Random walk with restarts

**Figure 2:**
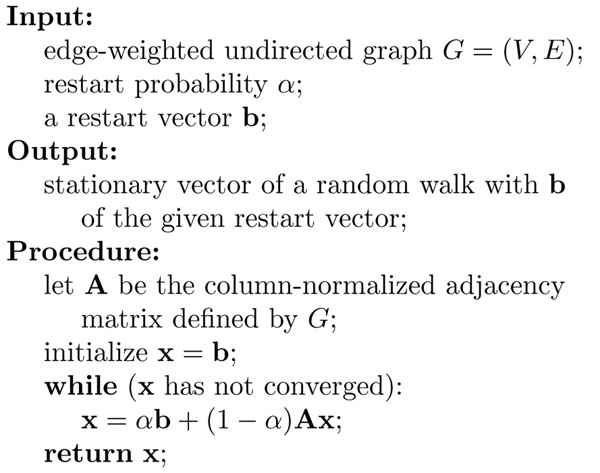
**Algorithm of a random walk with restarts** This pseudocode calculates the stationary vector of a random walk with restarts with a restart vector **b**.

The *random walk with restarts* or *repeated random walk* is a technique to find an affinity of start nodes *C* to all the individual nodes using a random walk. The algorithm for a random walk with restarts is given in Figure 2. We here describe a random walk with restarts with a cluster, *C,* of start nodes which are *equally weighted*[15-17], where the *i*-th element, *b_i_* of the restart vector, **b**, of the algorithm in Figure 2 is set to be

With this setting of **b**, the random walker, at every time tick, traverses from the current node to one of the adjacent node according to the weights of the corresponding edges, or goes back to one of the start nodes in *C* with probability  (i.e., the random walker chooses one of the start nodes in *C* uniformly at random ). The algorithm stops when the convergence condition *(e.g.,* the *L*_1_-norm between consecutive vectors x is less than 10^–10^) is satisfied. The result is a unique stationary vector. This vector can be considered to be the affinity or proximity from *C* to individual nodes. We denote the stationary vector by *p_C_,* and the *i*-th node, *v,* of *p_C_, i.e.,* the stationary probability from *C* to *v,* by *p_C_(v).* We also simply call it the *random walk distance from C to v.*

The restart probability *α* represents the degree of how a random walker can go far from the start nodes *C.* In other words, the closer to 1 *α* is, the more local structures around nodes of *C* the resulting stationary vector reflects. It should be noted here that when the restart probability *α* is smaller, the diameter of the neighborhood comprising of the visited nodes increases, and more iterations are needed to converge.

### Node-weighted expansion of clusters

Trivially, the algorithm in Figure 2 also works for a random walk with restarts at a single node, *i*. In this case, **b** is set to be a vector whose *i*-th element is one and zero for the others. Macropol *et al.*[17] proposed a protein complex prediction algorithm, RRW, which repeatedly expands a cluster, *C*, of nodes to a larger one by adding the node to which the random walk distance from *C* is the highest among all the nodes except ones in *C*. It can be easily showed that the stationary vector of a random walk with restarts with *C* is equivalent to the arithmetic mean of the stationary vectors of random walks with restarts at the single nodes in *C*, *i.e.,* for any node *v*,(1)

where *p_s_* is the stationary vector of a random walk with restarts at a node *s* (see [17,23,24] for proofs of this equation). Thus, the RRW algorithm efficiently computes the stationary vector of a random walk with restarts with *C* using Eq. (1), by precomputing the stationary vectors of random walks with restarts at all the single nodes on a given network. It should be noted here that this idea is equivalent to that of personalized PageRank in context-sensitive search on the Web [24-26]. The following example of **b** in context-sensitive search on the Web can be found in [24]: A user who wants to personalize on his bookmarked pages *C* uniformly would have a **b** where **b**(*s*) = 1*/*|*C*| if *s* ∈ *C*, and *u*(*s*) = 0 if *s* ∉ *C*. This is the same as in RRW.

**Figure 3:**
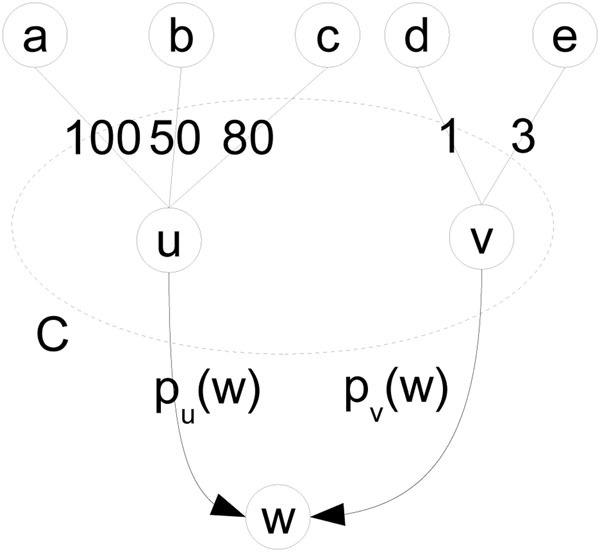
**A problem of expanding a cluster of nodes which are equally weighted** The ellipse with dashed line represents a cluster, denoted by *C*, which includes two nodes, *u* and *v*. Assume that *u* has three edges whose weights are 100, 50, 80, respectively, and *v* has two edges whose weights are 1 and 3, respectively. A node, *w*, is assumed to be out of *C*.

However, the method of a random walk with restarts with a cluster is problematic if the nodes of the cluster are equally weighted. Figure 3 gives an example of the problem. The ellipse with dashed line represents a cluster, denoted by *C*, which includes two nodes, *u* and *v*. Assume that *u* has three edges whose weights are 100, 50, 80, respectively, and *v* has two edges whose weights are 1 and 3, respectively. Namely, *u* is much richer than *v* in the supporting evidence for their interactions. Here consider the random walk distance from *C* to a node *w* which is out of *C*. In this case, the RRW algorithm takes the arithmetic mean of *p_u_*(*w*) and *p_v_*(*w*) (*i.e.*, the random walk distances from *u* and *v* to *w*, respectively) as *p_C_*(*w*). However, in this case, the arithmetic mean is not appropriate because *u* is much richer than *v* in the supporting evidence for their interactions. In other words, the random walk distance from *v* is not relatively reliable than that from *u*. This problem is clearly caused by the loss of the original PPI weights of a given PPI network. Thus, if the nodes of *C* were weighted adequately, a more likely new component to be added to *C* will be found. Then we will consider a way to assign to the restart vector **b** of the algorithm in Figure 2 a non-uniform vector.

We then extend the RRW algorithm by using a random walk with restarts with the cluster whose nodes are *non-uniformly weighted*. Suppose that we have a weight, *w_v_*, of a node *v* ∈ *V* . It is a separate issue how to determine the values of the weights, which will be discussed later. We then set the restart vector **b** as follows:

With this setting of **b**, a random walker, at every time tick, traverses from the current node to one of the adjacent node according to edge weights, or goes back to *i* ∈ *C* with the probability of

The next theorem shows that, even if nodes of a cluster are non-uniformly weighted, the stationary vector of a random walk with restarts with the cluster can be computed efficiently from the precomputed stationary vectors of random walks with restarts at the single nodes of the cluster. The proof is also trivial by the Linearity Theorem [23] as the case where the start nodes are equally weighted.

**Theorem 1***The stationary vector,***x***_C_, for a random walk with restarts starting with the set of weighted nodes, C, is*

**Figure 4:**
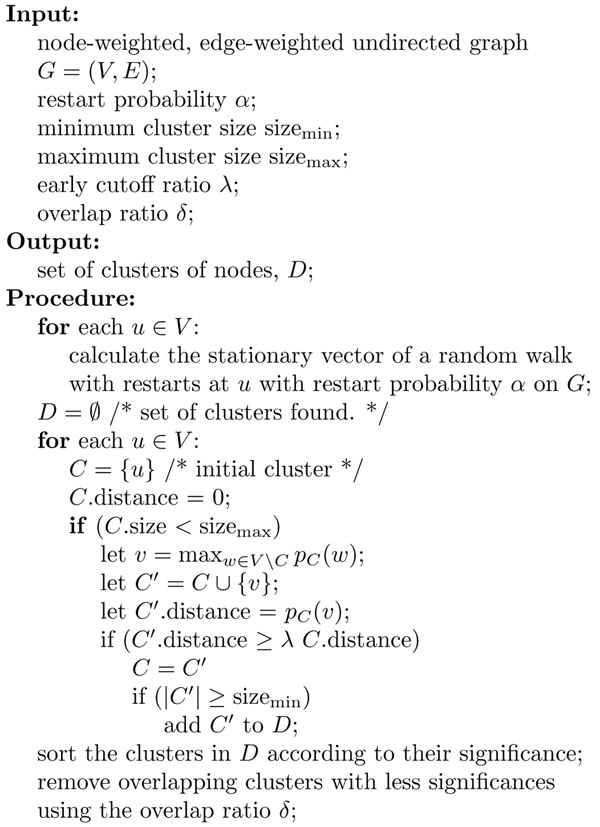
**Algorithm NWE** This pseudocode represents the overall algorithm, NWE, which is an extension of RRW. The difference between them is that NWE expands a current cluster, *C*, according to the stationary vector of a random walk with restarts with the cluster whose nodes are non-uniformly weighted. That stationary vector is represented by *p_C_*. On the other hand, RRW weights the nodes in *C* equally.

We next describe the overall algorithm of our method, shown in Figure 4. Notice that our algorithm, NWE, is almost the same as RRW. The difference between them is that NWE expands a current cluster according to the stationary vector of a random walk with restarts with the cluster whose nodes are non-uniformly weighted. Those nodes are equally weighted in RRW. We use the same statistical significance of a cluster, *C*, which is defined as

where *score*(*C*) is the score of *C*, which is the mean of all the random walk distances from a node in *C* to a node in *C*[17]. We also use the same overlap ratio between two clusters, *C*_1_ and *C*_2_, is defined as

If there are two clusters whose overlap ratio is greater than a specified threshold, the cluster with a less significance is removed.

### Matching statistics

In this work, we introduce *quantitative* matching statistics, called quantitative precision, recall, and f-measure, to evaluate a set of predicted clusters with known protein complexes. For two arbitrary sets of proteins, *s*_1_ and *s*_2_, which will be a pair of a predicted cluster and a known complex, the *concordance rate* between *s*_1_ and *s*_2_ is defined as

The rate is one if *s*_1_ and *s*_2_ are identical to each other and zero if any proteins are not shared by *s*_1_ and *s*_2_. Thus this statistic is a fairly rigorous index to see how much the two sets are similar to each other in size and membership of components because even if *s*_1_ is a proper subset of *s*_2_ the score is not optimal, and vice versa.

Let *C* be a set of predicted clusters and *K* a set of known complexes. The *quantitative cluster-wise precision* of a cluster *c ∈ C* with *K* is defined as

The quantitative complex-wise recall of a complex *k* ∈ *K* with *C* is defined as

The quantitative precision of *C* with *K* is defined as

where *C*′={*c* ∈ *C*|*precision _K_*(*c*) *>* 0}, and *Z_C'_ = ∑_c_*_∈_*_C′_* log |*c*|. Note that, a predicted cluster which does not overlap with any complexes in *K* is excluded because *K,* a reference set of known complexes, may be incomplete, i.e., not all the true protein complexes may be included in *K.* If predicted clusters which do not overlap with any known complexes were under consideration, the interpretation of the resulting value is not trivial. This issue is also mentioned by Brohée and van Helden [19].

Another feature of the definition of quantitative precision is that the quantitative precision is formulated as the weighted mean of non-zero quantitative cluster-wise precisions. It is mentioned by King *et al.*[11] that any overlap proportion of a small predicted cluster and a known complex is more likely to be by chance than the same overlap proportion involving a larger predicted cluster. In order to correct this problem, we have introduced the weighted mean of non-zero quantitative cluster-wise precisions, where the weight of a cluster is set to be proportional to the logarithm of the cluster size.

On the other hand, the quantitative recall of *C* is just the weighted mean of *all* known complexes in *K*. The *quantitative recall* of *C* with *K* is defined as

where *Z_K_* = ∑*_k∈K_* log |*k*|.

Using quantitative precision and recall, we define the *quantitative f-measure* of *C* and *K* as follows:

Note that the f-measure is the harmonic mean of precision and recall. For simplicity we omit the term “quantitative” from the quantitative measures defined above hereafter.

## Results and Discussion

We here report a performance comparison of our algorithm, NWE, with two existing algorithms, RRW, and MCL. The reason why MCL is selected here is that it is reported in the literature that MCL outperforms other clustering algorithms [19] and the Affinity Propagation algorithm [20]. RRW is selected because NWE is an extended algorithm of RRW. It is also a reason that all of the three algorithms can take as input an edge-weighted graph and exploit the weights to predict clusters.

### Node weight

The weight of a node is necessary for NWE, and a way to determine the value of it will be critical. However, in this work, we adopt the following simple way: We set the weight, *w_v_*, of a node, *v*, to be the total sum of the weights of all the edges adjacent to *v* in a given PPI network. This choice would be rational because of the example in Figure 3. If a more appropriate data source was available in the determination of node weights, it will contribute to more accurate predictions of our method.

### Parameter optimization of algorithms

Brohée and van Helden [19] carried out parameter optimization of four clustering algorithms, including MCL, with several randomized graphs derived from protein complexes of MIPS protein complex catalog [27], and compared performance of the algorithms with the optimized parameter sets of those algorithms. Note that edges of the graphs generated there are unweighted. This means that their scheme is not enough to our purpose.

We then extend their scheme by introducing a random assignment of weights to edges of those randomly generated graphs. An overview of the scheme we take here is as follows.

At first, in the same way as the work [19], we generate an underlying PPI graph, called the *test graph*, derived from MIPS protein complex catalog [27], which is a comprehensive catalog of manually curated protein complexes of *S. cerevisiae*. It contains 220 complexes, excluding complexes derived from high-throughput experimental data sets [3-5]. The list of those complexes can be found at the Brohée’s site [28]. The node set of the test graph consists of all the proteins belonging to some of the 220 protein complexes. Any pair of nodes within a single complex has an edge between them. The resulting graph is the test graph. It has 1 095 nodes and 14 343 edges.

In the next step, we derive from the test graph an altered graph by combining random edge deletions and additions on the test graph. The ratio of deleted edges, denoted by *del*, is set to either 40 or 80 percent. The ratio of added edges, denoted by *add*, is set to either 40 or 100 percent. Note that these percentages are w.r.t. the number of edges of the original test graph. As a result, we have four altered graphs. For a pair of *del* and *add*, we denote the resulting altered graph by *A_add, del_*.

Notice that an altered graph is edge-unweighted. It is required that an edge is weighted in our parameter optimization. A reasonable way to realize it is that, if an edge of an altered graph is also an original edge of the test graph, a relatively high weight is randomly assigned to the edge, and a relatively low weight is randomly assigned to the edge otherwise. We then take the following procedures. We prepare two uniform distributions on the intervals [*u*_min_, 100] and [1*, u*_max_], respectively, where *u*_min_ and *u*_max_ are integers in the interval [1, 100]. These distributions are denoted by UD_high_ and UD_low_, respectively. For an altered graph, *A_add, del_*, if an edge is also one existing in the test graph, an integer sampled from UD_high_ is assigned, and an integer sampled from UD_low_ is assigned otherwise. This procedure is applied to each of the four altered graphs with each combination of *u*_min_ = 30,70 and *u*_max_ = 30,70. As a result, we obtain a series of 16 further altered graphs. We denote these resulting graphs by *A*_*add, del, u*_min___*, u*_max__.

We next describe the search space of parameter sets of algorithms, which is shown in Table 1. The search spaces of parameter sets of NWE and RRW are determined based on the default parameter set of RRW. MCL is optimized in the inflation parameter, whose default value is 2.0.

**Table 1:** Optimal parameter sets of algorithms

Algorithm	Parameter	Search range	Optimal value
NWE	restart probability	0.6,0.7,0.8	0.6
	early cutoff	0.5, 0.6, 0.7	0.5
	overlap ratio	0.1, 0.2, 0.3	0.3
	minimum cluster size	4	4
	maximum cluster size	11, 50, 100	100

RRW	restart probability	0.6,0.7,0.8	0.6
	early cutoff	0.5, 0.6, 0.7	0.5
	overlap ratio	0.1, 0.2, 0.3	0.3
	minimum cluster size	4	4
	maximum cluster size	11, 50, 100	100

MCL	inflation	1.1, 1, 2, ⋯ , 3.0	1.6

The search spaces of parameter sets of NWE and RRW are determined based on the default parameter set of RRW [17]. MCL is optimized in the inflation parameter, whose default value is 2.0. All the combination of values of the parameters listed is evaluated.

What we should discuss here is the minimum size of predicted clusters and known complexes which we consider in computational experiments. It is known that any overlap proportion between a *small* predicted cluster (known complex, resp.) and a known complex (predicted cluster, resp.) is more likely to be by chance than the same overlap proportion involving a *larger* known complex (predicted cluster, resp.) [11,12]. We then consider clusters and complexes of size 4 and above, since the minimum size of clusters and complexes is often set to be four (see, for example, [12]). This is the reason why the minimum size of predicted clusters by NWE and RRW is set to be four (see Table 1). MCL has no option to set the minimum size of predicted clusters. Note that the number of protein complexes in the MIPS catalog is 118, which are used as gold standards in this parameter optimization.

All the combination of values of the parameters listed in Table 1 is considered and evaluated on the 16 altered graphs, *A*_*add, del, u*_min___*, u*_max__. For each parameter set, the mean of the f-measures on the 16 graphs is calculated. The parameter set with the optimal mean f-measure is adopted for further studies. Those optimal parameter sets are shown in Table 1. Notice that the optimal parameter set of NWE is the same as that of RRW.

Lastly, we must mention the parameter optimization of RRW. Instead of the software of RRW given by [17], we used our implementation of RRW, whose output is nearly the same as that of RRW. The reason is that the original RRW software has no function to save the result of random walk with restarts at single nodes. On the other hand, our program always saves the result if it is not stored in a specified directory, and reload it whenever the same input network, convergence threshold, and restart probability are given again. Thus, this function is very helpful to repeatedly execute an algorithm on the same input network with huge different sets of parameters. Actually, we have to execute RRW 1 296 times due to 16 altered graphs and 81 parameter sets. We were able to save much time by this strategy. Note that we used only the original RRW in further studies.

### Performance comparison on WI-PHI

With the optimal parameter sets determined in the previous section, the three algorithms are executed on the PPI network derived from WI-PHI. Table 2 shows the resulting performance measures of the three algorithms as well as the total number of predicted clusters and the number of predicted clusters which overlap with a complex of CYC2008.

**Table 2:** Performance comparison of algorithms on WI-PHI

Algorithm	NWE	RRW	MCL
# clusters	462	871	372
# overlaps	204 (0.44)	421 (0.48)	171 (0.46)
Precision	**0.57**	0.40	0.37
Recall	0.71	**0.75**	0.48
F-measure	**0.63**	0.52	0.42

The row labeled with “# clusters” shows the total number of predicted clusters and the next row gives the number of predicted clusters which overlap with a complex of CYC2008. The number in parentheses is the ratio of the number of those overlapping clusters to the total number of predicted clusters. The following rows show precision, recall, and f-measure, respectively. The best performance in each row is highlighted in bold.

The row labeled with “# clusters” shows the total number of predicted clusters. Notice that even though NWE is just an extended algorithm of RRW and the same parameter set is applied to them, those numbers are unexpectedly different between NWE and RRW, which have 462 and 871, respectively. Namely, the number of predicted clusters by RRW is nearly twice as large as that of NWE. This fact implies that the method of node-weighted expansion has a significant impact on the behavior of the algorithms. The next row, labeled with “# overlaps,” shows the number of predicted clusters which overlap with a complex of CYC2008. We can see that the ratios of them are similar to those of the previous row.

The last three rows of Table 2 show precision, recall, and f-measure, respectively. The best value in each row is highlighted in bold. In precision, NWE has the best value, 0.57, which is 42.5 percent greater than that of RRW, 0.40, and 54 percent greater than that of MCL, 0.37. In recall, NWE has the second best value, 0.71. The best value is given by RRW, 0.75. However, since the value of NWE is 5.3 percent lesser than that of RRW, the difference between them is little. The value of NWE is still 48 percent greater than that of MCL, 0.48.

Summary of the above observations is as follows: (i) NWE predicted lesser clusters than RRW, (ii) NWE has a higher precision than RRW, and (iii) NWE has nearly the same recall as RRW. Since this summary, it turns that NWE better avoids wrong predictions of clusters than RRW.

In f-measure, NWE attains the best value, 0.63, which is 21 percent greater than that of RRW, and 50 percent greater than that of MCL. As a conclusion, NWE has an ability of doing more balanced prediction than RRW. As a result, the introduction of the method of node-weighted expansion achieves significant and essential improvement to the algorithm of RRW.

The output of NWE with the optimal parameter set is shown in Additional file 1.

### Performance comparison on randomly shuffled graphs of WI-PHI

We next examine how robust the algorithms is to noise. We make a series of noisy networks from the WI-PHI network by the following way. The initial network is the original network derived from WI-PHI. A current network is repeatedly modified by shuffling edges of the network while preserving the degree of each node. For an integer *k* = 10, 20, 30, 40, the number of times of shuffling edges is set to be *k* percent of the number of interactions (50 000) of the original WI-PHI network. We thus have four types of noisy networks w.r.t. shuffling ratio. For each type, 10 random networks are generated. Performance measures of precision, recall, and f-measure are averaged over those 10 networks.

**Figure 5:**
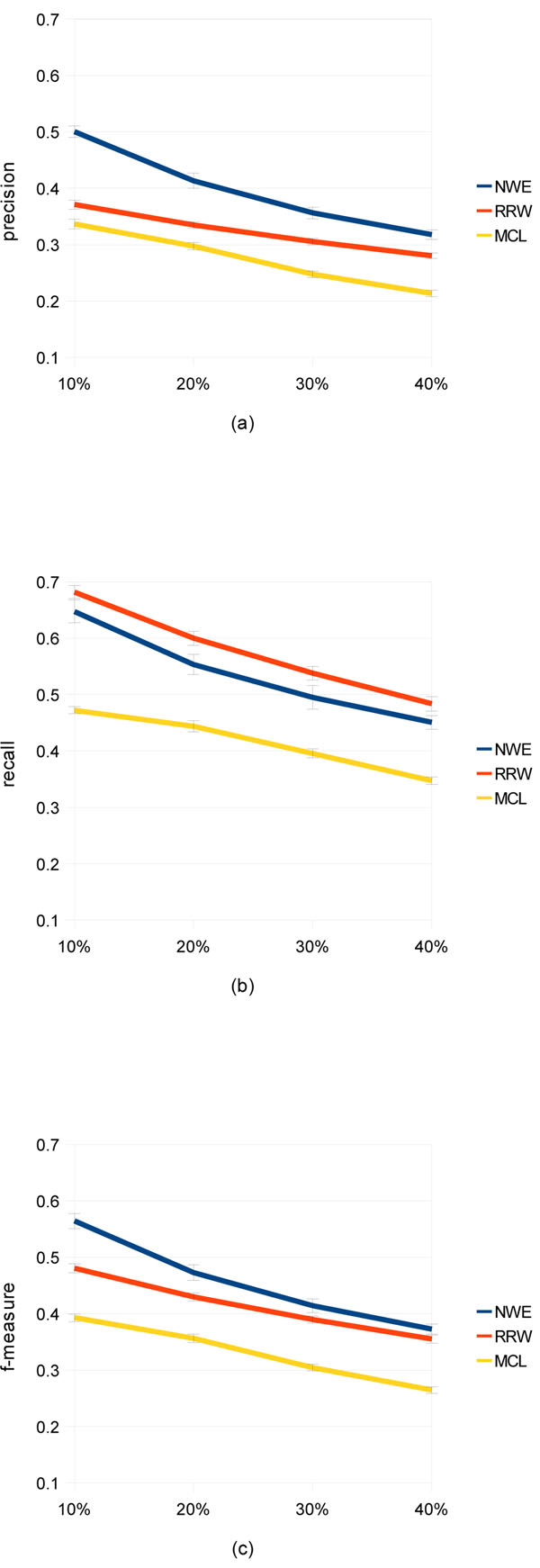
**Performance comparison on randomly shuffled graphs** (a), (b), and (c) show quantitative precision, recall, and f-measure, respectively. Input PPI networks are 10, 20, 30, and 40% edge-shuffled graphs of the network derived from WI-PHI. With each of the shuffle ratios, 10 shuffled graphs are generated randomly, on which performance is measured. An error bar shows the standard deviation.

Figure 5 shows how much noise can affect performance of the three algorithms. Note that the parameter sets used here is again the ones determined in the parameter optimization. It can be seen that the ranking of the algorithms is unchanged from the case with the original WI-PHI network, in each of precision, recall, and f-measure. In addition, we can observe that all of precision, recall, and f-measure decrease with increasing shuffling ratio. This result implies that the quality of a PPI network is very important for algorithms aggressively utilizing the weights of edges.

### Semantic homogeneity of predicted complexes

Lastly, we examine the richness of a GO term within a cluster to see the quality of predicted clusters, especially predicted clusters which have no overlap with any of the known complexes in CYC2008. It is useful to identify the GO term shared by most of the proteins within a cluster in order to characterize the cluster. We use the terms in a GO slim, instead of the whole GO. A GO slim is cut down to a subset of the terms in the whole GO. The purpose of it is to give a broad overview of the ontology content without the detail of the specific fine grained terms (see the site [29] for details of them). We here use the mapping of all yeast gene products to a GO-Slim term compiled by the SGD (*Saccharomyces* Genome Database) project. A file of the mapping is downloadable at the site [30]. The version of the file we used is dated March 26 2011.

**Figure 6:**
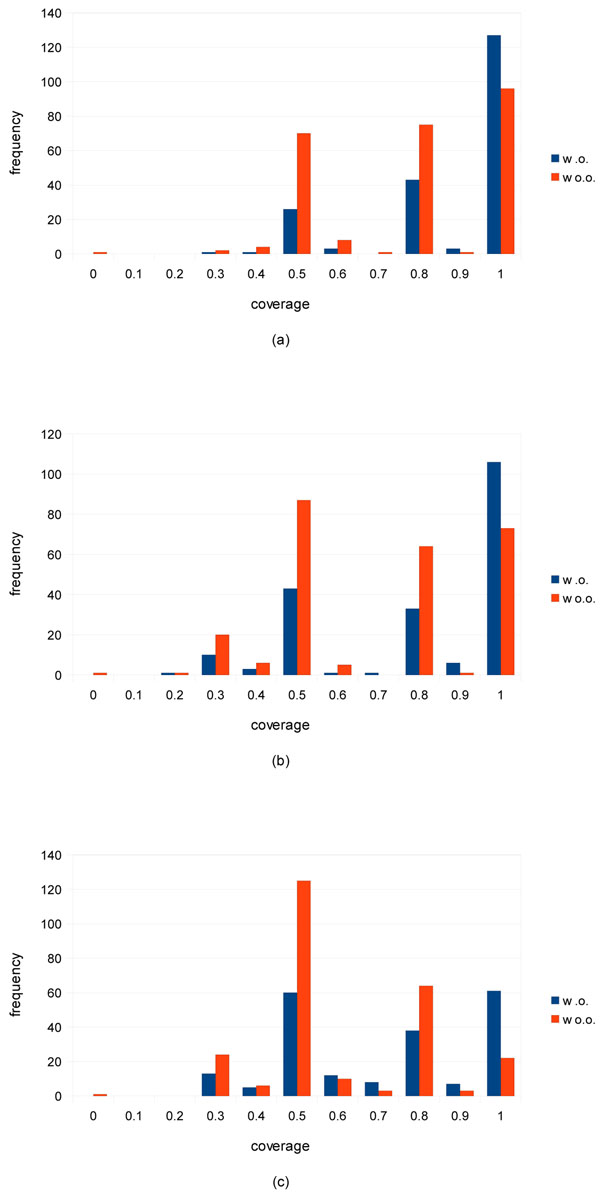
**Frequency distribution of coverages of predicted clusters by GO slim terms** The graphs, (a), (b), and (c) show the frequency distribution of coverages of predicted clusters over the yeast GO slim terms of the ontologies of cellular component, biological process, and molecular function, respectively. The “w.o.” bars represent the frequencies of coverage of predicted clusters with overlaps with some CYC2008 complexes. The “wo.o.” bars represent the frequencies of coverage of predicted clusters without any overlaps with all CYC2008 complexes. A real, *r*, on the horizontal axis indicates the range of coverage, (*r* – 0.1, *r*].

For a cluster, *C*, and a GO term, *t*, the *coverage* of *C* by *t* is defined as the ratio of the number of proteins in *C* annotated with *t* to the size of *C*. The *coverage* of *C* over an ontology of a GO slim is defined as the maximum of the coverages of *C* by the terms of the ontology of the GO slim. For each of the ontologies, cellular component, biological process, and molecular function, the frequency distribution of the coverages of predicted clusters over the yeast GO slim is shown in Figure 6. Note that predicted clusters we use here are ones that are generated by NWE with the optimal parameter set on the PPI network derived from WI-PHI.

Figure 6(a) shows the frequency w.r.t. the cellular component ontology. We can see in the graph that there are three peaks in the coverage ranges, (0.4, 0.5], (0.7,0.8], and (0.9, 1.0], respectively. The reason is as follows: The majority size of predicted clusters is four. Actually, 76.8 percent (355) of all the 462 predicted clusters are of size four. As a result, the possible coverages of a cluster of size four are 1.0, 0.75, 0.5, 0.25, and 0. Roughly speaking, in any coverage range, both bars do not make much difference in the heights. In the range of (0.9, 1.0], there are 127 and 96 predicted clusters which do and do not overlap with the protein complexes in CYC2008. Since they have high coverages, it can be expected that many of them form the whole or part of individual protein complexes. Actually, we can easily show such an example. A protein cluster consisting of VTI1/YMR197C, SYN8/YAL014C, YKT6/YKL196C, and PEP12/YOR036W is predicted by NWE of statistical significance 0.05. This cluster does not overlap with any of the CYC2008 protein complexes including ones of sizes two and three. The four proteins share the GO slim term, “cytoplasm.” Actually, VTI1/YMR197C and YKT6/YKL196C are directly annotated with the GO term, “SNARE complex,” which is a protein complex involved in membrane fusion.

Figure 6(b), which is the frequency w.r.t. the biological process ontology, looks nearly the same as (a). Thus, we can expect comparable results. For example, all the proteins of the predicted cluster taken above as an example share the GO slim term, “transport.” Figure 6(c), shows the frequency w.r.t. the molecular function ontology. This graph looks slightly different from the above two graphs. The bars in the range (0.9, 1.0] get lower and those in (0.4, 0.5] get higher.

This difference is also reflected in the Pearson correlation coefficience between the statistical significances of all the 462 predicted clusters and their coverage values over each ontology of cellular component, biological process, and molecular function, shown in Table 3. We have obtained the following interesting results. By the definition of the statistical significance, the negative sign of a Pearson correlation coefficience means a positive relationship between the statistical significance and the coverage. Here, a positive relationship means that a lower statistical significance corresponds to a higher coverage. Thus, the coverages over the biological process and molecular function ontologies have positive relationships with the statistical significance, though the coverage over the cellular component ontology unexpectedly does not. We also calculated the p-value of the t-test of a Pearson correlation coefficience on both sides, in which the null hypothesis is that the population correlation coefficient is zero. As can be seen, the Pearson correlation coefficience with the molecular function ontology is strongly significant. As a result, the null hypothesis can be rejected at appropriate significance levels, like 0.01. However, the remaining correlation coefficiences are not significant with any practical significance level.

**Table 3:** Pearson correlation coefficient and p-value

	CC	BP	MF
Correlation coefficient	0.051	-0.051	-0.25
P-value	0.28	0.27	7.4e-08

It is shown the Pearson correlation coefficience between the statistical significances of all the 462 predicted clusters and their coverages over each ontology of cellular component (CC), biological process (BP), and molecular function (MF). It is also given the p-value of the t-test of a Pearson correlation coefficience on both sides in which the null hypothesis is that the population correlation coefficient is zero.

We can consider a few reasons of this result. First of all, the GO is an ongoing project. The above correlation coefficients can be higher with more sophisticated GO annotations. The next reason is related to the GO term set we used, which is the GO slim of yeast. The semantic level of them can be a bit too general. Perhaps we should use the whole GO and calculate some semantic similarity of predicted clusters. Lastly, the statistical significance of a predicted cluster might be too simple because it is essentially the product of the size of a cluster and the mean of the single-start-node random walk distances between the nodes within the cluster. It may have room to improve.

## Conclusions

Our motivation of this work was to devise a protein complex prediction algorithm that can exploit the original PPI weights of WI-PHI as much as possible. We have introduced a way of assigning a weight to a node using the PPI weights of WI-PHI, and a random walk with restarts with a cluster of nodes whose are *non-uniformly weighted*. Our algorithm, NWE, expands a current cluster to a larger one by adding the node to which the resulting stationary probability from the cluster is the highest to the current cluster. In addition, we have also formulated fairly rigorous performance measures, quantitative precision, recall, and f-measure in order to evaluate biological significance of predicted clusters more accurately.

In performance comparison of NWE with RRW and MCL, NWE performs better than the others, even on noisy input networks, where the gold standards are manually curated heteromeric protein complexes of yeast in the CYC2008 database. Thus we can conclude that the node-weighted expansion method yields improvement in protein complex prediction. We have also examined the coverage of a predicted cluster by a GO term. The result shows that even a predicted cluster which does not overlap with any known complexes in CYC2008 is often covered widely by a common GO term. Thus, the predicted clusters are expected to be true protein complexes to some extent.

## Competing interests

The authors declare that they have no competing interests.

## Authors contributions

OM designed the computational methods, implemented the computer programs, performed most of the computational experiments, analyzed the results, and drafted and revised the paper. AC took part in the implementation and the computational experiments. Both authors read and approved the final manuscript.

## Supplementary Material

Additional file 1 **NWE output** This file contains the output of NWE with the parameter set determined in the parameter optimization. Each line corresponds to a predicted cluster of proteins, whose statistical significance is attached at the end. Click here for file
